# Generalized Dynamic Analytical Model of Piezoelectric Materials for Characterization Using Electrical Impedance Spectroscopy

**DOI:** 10.3390/ma12162502

**Published:** 2019-08-07

**Authors:** Hector de Castilla, Pierre Bélanger, Ricardo J. Zednik

**Affiliations:** Department of Mechanical Engineering, École de Technologie Supérieure, Université du Québec, Montréal, QC H3C1K3, Canada

**Keywords:** piezoelectricity, analytical model, impedance spectroscopy, electromechanical impedance spectroscopy

## Abstract

Piezoelectric materials have the intrinsic reversible ability to convert a mechanical strain into an electric field and their applications touch our daily lives. However, the complex physical mechanisms linking mechanical and electrical properties make these materials hard to understand. Computationally onerous models have historically been unable to adequately describe dynamic phenomena inside real piezoelectric materials, and are often limited to over-simplified first-order analytical, quasi-static, or unsatisfying phenomenological numerical approaches. We present a generalized dynamic analytical model based on first-principles that is efficiently computable and better describes these exciting materials, including higher-order coupling effects. We illustrate the significance of this model by applying it to the important 3m crystal symmetry class of piezoelectric materials that includes lithium niobate, and show that the model accurately predicts the experimentally observed impedance spectrum. This dynamic behavior is a function of almost all intrinsic properties of the piezoelectric material, so that material properties, including mechanical, electrical, and dielectric coefficients, can be readily and simultaneously extracted for any size crystal, including at the nanoscale; the only prior knowledge required is the crystal class of the material system. In addition, the model’s analytical approach is general in nature, and can increase our understanding of traditional and novel ferroelectric and piezoelectric materials, regardless of crystal size or orientation.

## 1. Introduction

Understanding the dynamic properties of a piezoelectric material that is subjected to mechanical vibrations or alternating electrical fields is of great importance: most real applications require dynamic solicitations at varying frequencies [1,2,3,4,5]. Presently, electrical impedance spectroscopy (EIS), in a piezoelectric context also referred to as electromechanical impedance spectroscopy (EMIS), is widely used to characterize piezoelectric and ferroelectric materials [6,7,8,9]. In this method, the material is subjected to an alternating voltage signal of varying frequency; this signal is converted by the material into a mechanical strain, which in turn is converted into a voltage response that can be measured. This makes this technique particularly useful in cases where the piezoelectric material has a novel geometry or is at the nanoscale, as becoming increasingly common in modern applications. The impedance spectrum thus obtained contains rich information on the chemical, mechanical, and electrical properties—the challenge being correctly extracting this information. In order to extract the material properties, the impedance spectrum, including resonance frequencies, must be accurately modeled. This model is compared to the experimental impedance spectrum and material parameters, including electromechanical coefficients, thereby determined [10,11,12,13].

However, the accuracy of this analysis is directly dependent on the quality of the model. Unfortunately, existing models are not suitable in many cases [14]. Attributing physical meaning to phenomenological numerical approaches, such as equivalent circuit analyses [15,16,17], is often challenging. Therefore analytical models are generally preferred, with the Butterworth Van-Dycke model being the most commonly employed [18]. However, this model is one dimensional and does not describe the coupling between multiple resonances or losses that would occur in a finite, real, sample. Improvements on this approach, the modified Butterworth Van-Dycke model [19] and the generalized Butterworth Van-Dycke model [20] take into account acoustic losses, dielectric losses, and losses in the electrodes, but still do not describe the coupling between two or more modes. In addition, all of these models make simplifying approximations from piezoelectric equations and do not predict harmonics. The most recent model, developed by Michel Brissaud in 2010 [21,22], uses an analytical method based on the basic equations of piezoelectricity. Although this model is the most accurate and complete available to date, only the three principal plane waves were considered. Thus, all shear modes and coupling with shear waves are not predicted. Moreover, many assumptions are needed, and the model is limited to the 6 mm crystal class of piezoelectric materials only. We therefore propose a more complete analytical model that greatly reduces the number of underlying assumptions, extends to mechanical shear waves [23], is applicable to any sample geometry or crystal symmetry, and is entirely based on first-principles.

## 2. Materials and Methods

**Figure 1:**
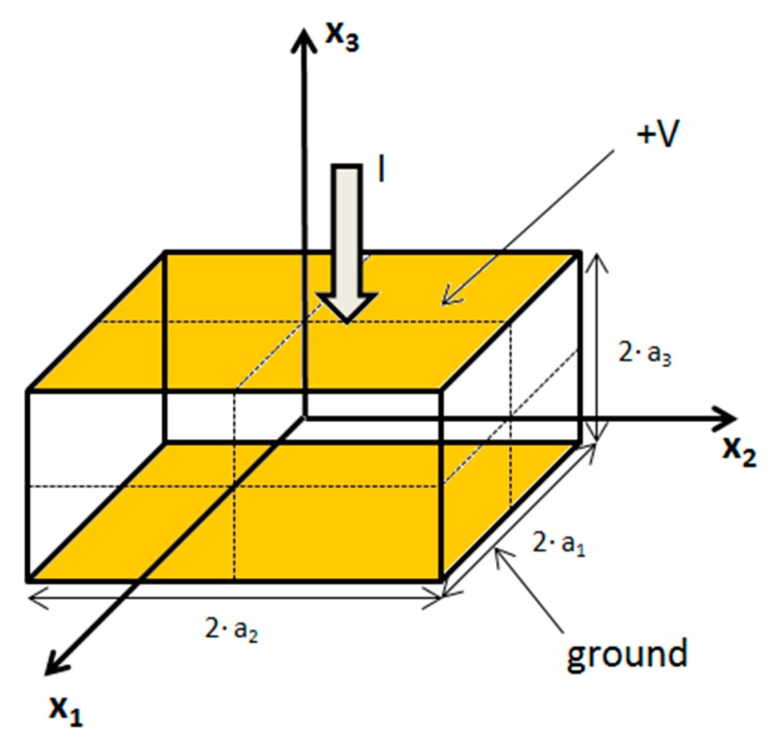
Axis orientation and conventions used in the model.

Consider a single crystal of any piezoelectric crystal class with a given stiffness, piezoelectric, and permittivity matrix. We assume, for sake of argument, that the sample is a rectangular cuboid in an orthonormal basis defined with three vectors $\vec{x_{1}}$, $\vec{x_{2}}$, $\vec{x_{3}}$, as shown in Figure 1, although in principle the model can accommodate any sample geometry. The symbols used to describe the model are summarized in Table 1. The dimensions of the piezoelectric material are 2·a_1_ along x_1_ axis, 2·a_2_ along x_2_ axis and 2·a_3_ along x_3_ axis. Two electrodes are on two parallel faces. Arbitrarily, the electrodes are assumed on faces normal to the x_3_ axis. They are assumed to be ideal conductors with a relative potential V on the top electrode and zero on the bottom electrode. The current I is defined in receptor convention between the electrodes. The sample is assumed mechanically free on its six faces.

Resonances in the sample are the result of standing waves due to a dynamic solicitation [24]. Those standing waves are the consequence of waves propagating along the x_1_, x_2_, and x_3_ axes. Indeed, these axes are the only directions that allow a reflected wave to superpose with its incident wave. Using Christoffel’s equations [25], there are three waves per direction (usually one longitudinal wave and two shear waves) propagating with three velocities. The linearity of the equations means that the mechanical displacement is a linear superposition of the nine standing waves. Thus, there are nine unknown parameters A_i,j_ to determine:

(1) $$U\left(x_{1}{,x}_{2}{,x}_{3},t\right)=\sum_{i=1}^{3}\left(A_{i,1}\cdot f_{i,1}\left(x_{1}\right){+A}_{i,2}\cdot f_{i,2}\left(x_{2}\right){+A}_{i,3}\cdot f_{i,3}{(x}_{3})\right)\cdot e^{j\cdot \omega \cdot t}.$$

from this form of displacement, the strain tensor can be easily deduced, using Einstein summation notation [26]:

(2) $$S_{\mathrm{ij}}=\frac{1}{2}\left(\frac{\partial u_{i}}{\partial x_{j}}+\frac{\partial u_{j}}{\partial x_{i}}\right),\;S=\left(\begin{array}{c} S_{11}\\ S_{22}\\ S_{33}\\ S_{23}\\ S_{13}\\ S_{12} \end{array}\right)=\left(\begin{array}{c} S_{1}\\ S_{2}\\ S_{3}\\ S_{4}\\ S_{5}\\ S_{6} \end{array}\right).$$

without loss of generality, S and E are arbitrarily chosen as independent variables in order to write the governing piezoelectric expressions [27] as:

(3) $$\{\begin{array}{c} {T=c}_{E}\cdot {S-e}^{t}\cdot E\\ D=e\cdot {S+\varepsilon }_{S}\cdot E \end{array}.$$

Since the metal electrodes are assumed to be perfect conductors, the electric potential is uniform throughout each electrode. Moreover, the electrical current is assumed to flow entirely through the piezoelectric crystal between the electrodes. The electrical boundary conditions are therefore given by:

(4a) $$\int_{{-a}_{1}}^{a_{1}}E_{1}{\mathrm{dx}}_{1}=0,$$

(4b) $$\int_{{-a}_{2}}^{a_{2}}E_{2}{\mathrm{dx}}_{2}=0,$$

(4c) $$\int_{{-a}_{3}}^{a_{3}}E_{3}{\mathrm{dx}}_{3}=-V,$$

(4d) $$\int_{{-a}_{2}}^{a_{2}}\int_{{-a}_{3}}^{a_{3}}D_{1}{\mathrm{dx}}_{2}{\mathrm{dx}}_{3}=0,$$

(4e) $$\int_{{-a}_{1}}^{a_{1}}\int_{{-a}_{3}}^{a_{3}}D_{2}{\mathrm{dx}}_{2}{\mathrm{dx}}_{3}=0,$$

(4f) $$\int_{{-a}_{1}}^{a_{1}}\int_{{-a}_{2}}^{a_{2}}D_{3}{\mathrm{dx}}_{1}{\mathrm{dx}}_{2}=Q.$$

The free surfaces mean that the stresses normal to the six faces of the sample are zero. The mechanical boundary conditions are therefore given by:

(5a) $$\forall x_{2}{,\;x}_{3}{,\;x}_{1}=\left\{{-a}_{1}{,a}_{1}\right\}{,\;T}_{1}=0,$$

(5b) $$\forall x_{1}{,\;x}_{3}{,\;x}_{2}=\left\{{-a}_{2}{,a}_{2}\right\}{,\;T}_{2}=0,$$

(5c) $$\forall x_{1}{,\;x}_{2}{,\;x}_{3}=\left\{{-a}_{3}{,a}_{3}\right\}{,\;T}_{3}=0,$$

(5d) $$\forall x_{1}{,\;x}_{3}{,\;x}_{2}=\left\{{-a}_{2}{,a}_{2}\right\}{,\;T}_{4}=0,$$

(5e) $$\forall x_{1}{,\;x}_{2}{,\;x}_{3}=\left\{{-a}_{3}{,a}_{3}\right\}{,\;T}_{4}=0,$$

(5f) $$\forall x_{2}{,\;x}_{3}{,\;x}_{1}=\left\{{-a}_{1}{,a}_{1}\right\}{,\;T}_{5}=0,$$

(5g) $$\forall x_{1}{,\;x}_{2}{,\;x}_{3}=\left\{{-a}_{3}{,a}_{3}\right\}{,\;T}_{5}=0,$$

(5h) $$\forall x_{2}{,\;x}_{3}{,\;x}_{1}=\left\{{-a}_{1}{,a}_{1}\right\}{,\;T}_{6}=0,$$

(5i) $$\forall x_{1}{,\;x}_{3}{,\;x}_{2}=\left\{{-a}_{2}{,a}_{2}\right\}{,\;T}_{6}=0.$$

The electric field and the dielectric displacement field at a specific point of the material are unknown. It is therefore necessary to rewrite the above equations with only V and Q instead of E and D. The piezoelectric Equations (3) therefore need to be integrated along each face of the sample in order to satisfy the mechanically boundary conditions. For example, the equation of the first stress component becomes:

(6) $$\int_{{-a}_{2}}^{a_{2}}\int_{{-a}_{3}}^{a_{3}}T_{1}\left(a_{1}\right){\mathrm{dx}}_{3}{\mathrm{dx}}_{2}=0=\int_{{-a}_{2}}^{a_{2}}\int_{{-a}_{3}}^{a_{3}}c_{E}\cdot S\left(a_{1}\right){\mathrm{dx}}_{3}{\mathrm{dx}}_{2}+{\left(e_{k,1}\right)}^{t}\cdot \int_{{-a}_{2}}^{a_{2}}\int_{{-a}_{3}}^{a_{3}}\left(\begin{array}{c} E_{1}(a_{1})\\ E_{2}(a_{1})\\ E_{3}(a_{1}) \end{array}\right){\mathrm{dx}}_{3}{\mathrm{dx}}_{2},$$

(7) $$\int_{{-a}_{2}}^{a_{2}}\int_{{-a}_{3}}^{a_{3}}\left(\begin{array}{c} E_{1}\left(a_{1}\right)\\ E_{2}\left(a_{1}\right)\\ E_{3}\left(a_{1}\right) \end{array}\right){\mathrm{dx}}_{3}{\mathrm{dx}}_{2}=\left(\begin{array}{c} \int_{{-a}_{2}}^{a_{2}}\int_{{-a}_{3}}^{a_{3}}E_{1}\left(a_{1}\right){\mathrm{dx}}_{3}{\mathrm{dx}}_{2}\\ 0\\ -2{\cdot a}_{2}\cdot V \end{array}\right),\;k=\left\{1,2,3\right\}.$$

Explicitly, E_1_ can be expressed as a function of D_1_ thanks to the second piezoelectric equation:

(8) $$\begin{array}{c} \overset{a_{2}}{\underset{{-a}_{2}}{\int }}\overset{a_{3}}{\underset{{-a}_{3}}{\int }}\left(\begin{array}{c} E_{1}\\ E_{2}\\ E_{3} \end{array}\right){\mathrm{dx}}_{3}{\mathrm{dx}}_{2}=\left(\begin{array}{c} \varepsilon_{S_{1,1}}^{-1}\cdot \overset{a_{2}}{\underset{{-a}_{2}}{\int }}\overset{a_{3}}{\underset{{-a}_{3}}{\int }}{(D}_{1}{-e}_{1,j}\cdot {S(a}_{1}{))\mathrm{dx}}_{3}{\mathrm{dx}}_{2}\\ 0\\ -2\cdot a_{2}\cdot V \end{array}\right)\\ =\left(\begin{array}{c} \varepsilon_{S_{1,1}}^{-1}\cdot e_{1,j}\cdot \int_{{-a}_{2}}^{a_{2}}\int_{{-a}_{3}}^{a_{3}}S\left(a_{1}\right){\mathrm{dx}}_{3}{\mathrm{dx}}_{2}\\ 0\\ -2\cdot a_{2}\cdot V \end{array}\right),\;j=\left\{1,2,3,4,5,6\right\}, \end{array}$$

(9) $$\int_{{-a}_{2}}^{a_{2}}\int_{{-a}_{3}}^{a_{3}}c_{E}\cdot S\left(a_{1}\right){\mathrm{dx}}_{3}{\mathrm{dx}}_{2}+{\left(e_{k,1}\right)}^{t}\cdot \left(\begin{array}{c} \varepsilon_{S_{1,1}}^{-1}\cdot e_{1,j}\cdot \int_{{-a}_{2}}^{a_{2}}\int_{{-a}_{3}}^{a_{3}}{S(a}_{1}{)\mathrm{dx}}_{3}{\mathrm{dx}}_{2}\\ 0\\ -2\cdot a_{2}\cdot V \end{array}\right)=0.$$

Similarly, using all nine mechanical boundaries conditions (Equations (5a–i)), a system representing the displacement field with nine equation and nine unknowns is obtained. The solution gives the expression of the unknowns as a function of V, Q, the frequency, and the intrinsic parameters of the material. The displacement field is now well described.

According to the crystal class and the component considered, the following piezoelectric stress equations can be used to simplify calculations:

(10) $${T=C}_{D}\cdot {S-h}^{t}\cdot D,$$

(11) $$C_{D}{=C}_{E}+e^{t}\cdot \varepsilon_{S}^{-1}\cdot e,$$

(12) $${h=\varepsilon }_{S}^{-1}\cdot e.$$

The second piezoelectric Equation (3) must be used to obtain the impedance spectrum. To satisfy electrical boundaries conditions, it is necessary to integrate the equation over the entire volume of the sample:

(13) $$\int_{{-a}_{1}}^{a_{1}}\int_{{-a}_{2}}^{a_{2}}\int_{{-a}_{3}}^{a_{3}}T_{1}\left(\begin{array}{c} D_{1}\\ D_{2}\\ D_{3} \end{array}\right){\mathrm{dx}}_{1}{\mathrm{dx}}_{2}{\mathrm{dx}}_{3}=e\cdot \int_{{-a}_{1}}^{a_{1}}\int_{{-a}_{2}}^{a_{2}}\int_{{-a}_{3}}^{a_{3}}S{\mathrm{dx}}_{1}{\mathrm{dx}}_{2}{\mathrm{dx}}_{3}+\varepsilon_{S}\cdot \int_{{-a}_{1}}^{a_{1}}\int_{{-a}_{2}}^{a_{2}}\int_{{-a}_{3}}^{a_{3}}\left(\begin{array}{c} E_{1}\\ E_{2}\\ E_{3} \end{array}\right){\mathrm{dx}}_{1}{\mathrm{dx}}_{2}{\mathrm{dx}}_{3}.$$

In order to have a relation between V and Q, only the third component of the equation is pertinent, so that:

(14) $$2\cdot a_{3}\cdot {Q=e}_{3,j}\cdot \int_{{-a}_{1}}^{a_{1}}\int_{{-a}_{2}}^{a_{2}}\int_{{-a}_{3}}^{a_{3}}{\mathrm{Sdx}}_{1}{\mathrm{dx}}_{2}{\mathrm{dx}}_{3}{-\varepsilon }_{S_{3,3}}\cdot 4\cdot a_{1}\cdot a_{2}\cdot V.$$

We now have a generalized analytical model that describes the behavior of any piezoelectric material, regardless of crystal symmetry.

## 3. Results and Discussion

### 3.1. Application to the 3m

In order to better illustrate the significance of the general dynamic analytical model derived above, we apply it to the 3m crystal class, one of the most challenging crystal symmetries. The 3m crystal symmetry class is a trigonal point group corresponding to several common piezoelectric materials, including lithium niobate [28]. Due to the multifold symmetry of the 3m crystal class, these types of materials have notoriously complex impedance spectra, with numerous resonances and coupled modes, even in a sample with simple geometry [29,30]. Conventional models are unable to predict most resonances and therefore fail to accurately predict the impedance spectrum [31,32].

In this illustration, we consider a rectangular sample oriented along the basic Cartesian directions (x, y, z). The electrodes are on faces normal to the z-axis. For the 3m crystal class, the mechanical stiffness matrix in a constant electric field, the piezoelectric matrix, and the permittivity matrix are, respectively, given by [33]

(15) $$C_{E}=(\begin{array}{cccccc} c_{E_{11}} & c_{E_{12}} & c_{E_{13}} & c_{E_{14}} & 0 & 0\\ c_{E_{12}} & c_{E_{11}} & c_{E_{13}} & -c_{E_{14}} & 0 & 0\\ c_{E_{13}} & c_{E_{13}} & c_{E_{33}} & 0 & 0 & 0\\ c_{E_{14}} & -c_{E_{14}} & 0 & c_{E_{44}} & 0 & 0\\ 0 & 0 & 0 & 0 & c_{E_{44}} & c_{E_{14}}\\ 0 & 0 & 0 & 0 & c_{E_{14}} & c_{E_{66}} \end{array}),$$

(16) $$e=\left(\begin{array}{cccccc} 0 & 0 & 0 & 0 & e_{15} & {-e}_{22}\\ {-e}_{22} & e_{22} & 0 & e_{15} & 0 & 0\\ e_{31} & e_{31} & e_{33} & 0 & 0 & 0 \end{array}\right),$$

(17) $$\varepsilon_{S}=\left(\begin{array}{ccc} \varepsilon_{S_{11}} & 0 & 0\\ 0 & \varepsilon_{S_{11}} & 0\\ 0 & 0 & \varepsilon_{S_{33}} \end{array}\right).$$

As shown above, the first step in applying our general dynamic analytical piezoelectric model requires determining the form of the displacement field from Christoffel’s equations. The waves propagating with velocity v along a unit vector (x_1_, x_2_, x_3_) are a solution to the following equation:

(18) $$L\cdot C\cdot L^{t}\cdot {U=-\rho v}^{2}U,$$

(19) $$L=\left(\begin{array}{cccccc} x_{1} & 0 & 0 & 0 & x_{1} & x_{1}\\ 0 & x_{2} & 0 & x_{2} & 0 & x_{2}\\ 0 & 0 & x_{3} & x_{3} & x_{3} & 0 \end{array}\right),$$

(20) $$U=\left(\begin{array}{c} u_{1}\\ u_{2}\\ u_{3} \end{array}\right).$$

where U is the displacement, C the effective stiffness of the material, and ρ the density of the material. The effective stiffness matrix C is different from C_E_ or even C_D_, as C is the stiffness of the sample with ideal electrodes and neglecting losses; a generalization that takes into account losses is presented later in the manuscript. With the electrodes on faces normal to a unit vector (x_1_, x_2_, x_3_),

(21) $${C=C}_{E}{+e}^{t}\cdot \left(\varepsilon_{S}^{-1}\cdot \left(\begin{array}{ccc} x_{1} & 0 & 0\\ 0 & x_{2} & 0\\ 0 & 0 & x_{3} \end{array}\right)\right)\cdot e.$$

The eigenvectors and the eigenvalues of the Christoffel equation describe all the waves propagating along a certain direction. For the 3m crystal class, we therefore find that:

(22) $$C=(\begin{array}{cccccc} c_{E_{11}}+\frac{e_{31}^{2}}{\varepsilon_{S_{33}}} & c_{E_{12}}+\frac{e_{31}^{2}}{\varepsilon_{S_{33}}} & c_{E_{13}}+\frac{e_{31}\cdot e_{33}}{\varepsilon_{S_{33}}} & c_{E_{14}} & 0 & 0\\ c_{E_{12}}+\frac{e_{31}^{2}}{\varepsilon_{S_{33}}} & c_{E_{11}}+\frac{e_{31}^{2}}{\varepsilon_{S_{33}}} & c_{E_{13}}+\frac{e_{31}\cdot e_{33}}{\varepsilon_{S_{33}}} & -c_{E_{14}} & 0 & 0\\ c_{E_{13}}+\frac{e_{31}\cdot e_{33}}{\varepsilon_{S_{33}}} & c_{E_{13}}+\frac{e_{31}\cdot e_{33}}{\varepsilon_{S_{33}}} & c_{E_{33}}+\frac{e_{33}^{2}}{\varepsilon_{S_{33}}} & 0 & 0 & 0\\ c_{E_{14}} & -c_{E_{14}} & 0 & c_{E_{44}} & 0 & 0\\ 0 & 0 & 0 & 0 & c_{E_{44}} & c_{E_{14}}\\ 0 & 0 & 0 & 0 & c_{E_{14}} & c_{E_{66}} \end{array}),$$

(23) $$U\left(x,y,z,t\right)=\sum_{i=1}^{3}\left(A_{x,i}\cdot V_{x,i}\cdot \sin\left(\alpha_{x,i}\cdot x\right){+A}_{y,i}\cdot V_{y,i}\cdot \sin\left(\alpha_{y,i}\cdot y\right){+A}_{z,i}\cdot V_{z,i}\cdot \sin\left(\alpha_{z,i}\cdot z\right)\right)\cdot e^{j\omega t},$$

(24) $$\alpha_{x,i}=\frac{\omega }{v_{x,i}}.$$

where V_x,1_ is the first eigenvector of the Christoffel equation for the x direction, v_x,1_ is the associated velocity, ω is the pulsation, and A_x,1_ is the undetermined constant due to the linear superposition assumption. The next step is to determine these constants from the piezoelectric equations. Analogous to the general case above, the expression of the strain field is deduced from the displacement field, so that the system of nine equations to solve the nine unknowns becomes

(25a) $$\int_{{-a}_{2}}^{a_{2}}\int_{{-a}_{3}}^{a_{3}}T_{1}{\mathrm{dx}}_{3}{\mathrm{dx}}_{2}=0=\int_{{-a}_{2}}^{a_{2}}\int_{{-a}_{3}}^{a_{3}}c_{E}\cdot S\left(a_{1}\right){\mathrm{dx}}_{3}{\mathrm{dx}}_{2}{+e}_{31}\cdot 2\cdot a_{2}\cdot V,$$

(25b) $$\int_{{-a}_{1}}^{a_{1}}\int_{{-a}_{3}}^{a_{3}}T_{2}{\mathrm{dx}}_{3}{\mathrm{dx}}_{1}=0=\int_{{-a}_{1}}^{a_{1}}\int_{{-a}_{3}}^{a_{3}}c_{E}\cdot S\left(a_{2}\right){\mathrm{dx}}_{3}{\mathrm{dx}}_{1}{+e}_{31}\cdot 2\cdot a_{1}\cdot V,$$

(25c) $$\int_{{-a}_{1}}^{a_{1}}\int_{{-a}_{2}}^{a_{2}}T_{3}{\mathrm{dx}}_{1}{\mathrm{dx}}_{2}=0=\int_{{-a}_{1}}^{a_{1}}\int_{{-a}_{2}}^{a_{2}}c_{D}\cdot S\left(a_{3}\right){\mathrm{dx}}_{2}{\mathrm{dx}}_{1}{-h}_{33}\cdot Q,$$

(25d) $$\int_{{-a}_{1}}^{a_{1}}\int_{{-a}_{2}}^{a_{2}}T_{4}{\mathrm{dx}}_{2}{\mathrm{dx}}_{1}=0=\int_{{-a}_{1}}^{a_{1}}\int_{{-a}_{2}}^{a_{2}}c_{E}\cdot S\left(a_{3}\right){\mathrm{dx}}_{2}{\mathrm{dx}}_{1},$$

(25e) $$\int_{{-a}_{1}}^{a_{1}}\int_{{-a}_{3}}^{a_{3}}T_{4}{\mathrm{dx}}_{3}{\mathrm{dx}}_{1}=0=\int_{{-a}_{1}}^{a_{1}}\int_{{-a}_{3}}^{a_{3}}c_{D}\cdot S\left(a_{2}\right){\mathrm{dx}}_{3}{\mathrm{dx}}_{1},$$

(25f) $$\int_{{-a}_{1}}^{a_{1}}\int_{{-a}_{2}}^{a_{2}}T_{5}{\mathrm{dx}}_{2}{\mathrm{dx}}_{1}=0=\int_{{-a}_{1}}^{a_{1}}\int_{{-a}_{2}}^{a_{2}}c_{E}\cdot S\left(a_{3}\right){\mathrm{dx}}_{2}{\mathrm{dx}}_{1},$$

(25g) $$\int_{{-a}_{2}}^{a_{2}}\int_{{-a}_{3}}^{a_{3}}T_{5}{\mathrm{dx}}_{3}{\mathrm{dx}}_{2}=0=\int_{{-a}_{2}}^{a_{2}}\int_{{-a}_{3}}^{a_{3}}c_{D}\cdot S\left(a_{1}\right){\mathrm{dx}}_{3}{\mathrm{dx}}_{2},$$

(25h) $$\int_{{-a}_{1}}^{a_{1}}\int_{{-a}_{3}}^{a_{3}}T_{6}{\mathrm{dx}}_{3}{\mathrm{dx}}_{1}=0=\int_{{-a}_{1}}^{a_{1}}\int_{{-a}_{3}}^{a_{3}}c_{E}\cdot S\left(a_{2}\right){\mathrm{dx}}_{3}{\mathrm{dx}}_{1},$$

(25i) $$\int_{{-a}_{2}}^{a_{2}}\int_{{-a}_{3}}^{a_{3}}T_{6}{\mathrm{dx}}_{3}{\mathrm{dx}}_{2}=0=\int_{{-a}_{2}}^{a_{2}}\int_{{-a}_{3}}^{a_{3}}c_{D}\cdot S\left(a_{1}\right){\mathrm{dx}}_{3}{\mathrm{dx}}_{2}.$$

In order to simplify these expressions, the piezoelectric stress equation in a constant dielectric displacement field was used for Equations (3), (5), (7) and (9). Resolving this system of equations allows us to determine the displacement field as a function of the frequency, V, Q, the geometry, and the intrinsic properties of the material. In order to obtain the relationship between V and Q, the piezoelectric equation of the dielectric displacement (Equation (14)) is needed. Solving this equation, the impedance can be expressed as a function of the frequency, geometry, and intrinsic properties by:

(26) $$Z=\frac{V}{-j\cdot \omega \cdot Q}.$$

In a more generalized case, and for improved accuracy, when the impedance expression as a function of intrinsic properties, geometry and frequency is computed, it is also possible to take into account loss effects. The model can be generalized to include any number of loss mechanisms, including, but not limited to mechanical dampening, dielectric losses, ionic conductivity, etc.

To do so, coefficients of the material need to be substituted with complex expressions. Although such an operation improves the accuracy of the model, the merits should be carefully balanced with the consequence of an increased computational difficulty in fitting the experimental impedance spectrum. The mechanical damping of the crystal may be not negligible in certain conditions like high temperature, high frequency or for certain materials like piezoelectric polymers [34]. For example, in this case the stiffness matrix must include imaginary components to take into account the losses, and becomes:

(27) $$c_{E}=c_{E}^{\prime }-j\cdot c_{E}^{\prime \prime }{=c}_{E}^{\prime }-j\cdot \eta_{E}\cdot \omega .$$

At high temperature or high frequency, dielectric losses could also affect the impedance spectrum [35,36]. To take these into account, the permittivity matrix has to be substituted with

(28) $$\varepsilon =\varepsilon^{\prime }-j\cdot \varepsilon^{\prime \prime }.$$

Sometimes, an electrical conductivity σ may appear inside the sample due to ionic diffusion or other processes, especially at high temperature and low frequency [37]. Then, the permittivity matrix has to be substituted with

(29) $${\varepsilon =\varepsilon }^{\prime }-j\cdot \varepsilon^{\prime \prime }+\frac{\sigma }{j\cdot \omega }.$$

In all cases, taking into consideration these, or any other, piezoelectric loss mechanisms does not change the approach of the model, but does add some computational complexity. The ability to extend our model to include any loss mechanism of interest is of particular value to understand the underlying physical mechanisms in the piezoelectric material considered.

### 3.2. Experimental Illustration

In order to demonstrate how our analytical dynamic model compares to experimental observations, we take the model derived above for the 3m crystal class and use it to analyze lithium niobate, a technologically important piezoelectric material. For this experiment, we employed a Z-cut congruently grown single crystal of lithium niobate and a Y-cut congruently grown single crystal of lithium niobate; both crystals were cut from the same ingot grown by a proprietary Czochralski method and supplied by MTI Corporation, California. The geometry for both samples was a square of 10 mm width and 0.5 mm thickness, although in principle any arbitrary geometry could have been used. A 100 nm thick platinum electrode on a titanium dioxide adhesion layer was sputtered onto both complete sides of the crystal to produce a parallel plate metal-oxide-metal (MOM) capacitor structure (10 mm × 10 mm electrodes). The impedance spectrum was measured with a Keysight E4990A impedance analyzer at room temperature using platinum probes and a two-terminal measurement from 20 Hz to 10 MHz [36]. The datasets generated and analyzed during the current study are available from the corresponding author on reasonable request.

**Figure 2:**
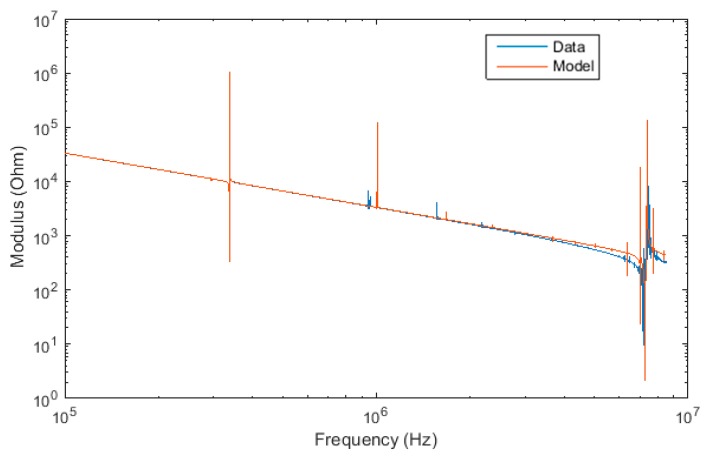
Model fitting on the impedance modulus spectrum of the Z-cut sample.

The model was analytically resolved using Matlab on an Intel^®^ Core™ i5-4300U processor for LiNbO_3_ in less than one hour. Once analytically resolved, numerically evaluating the analytical model to extract the material parameters from any given impedance spectrum was then realized in under 0.2 s. This compares favorably to conventional numerical models that can take hours or days to resolve on the same processor, depending on the complexity of the numerical model selected (e.g., FEA, equivalent circuit analysis, etc.). The apparent noise visible in the impedance spectrum at high frequency is the result of numerous small resonances. Indeed, the densities of resonances increase with the frequency, due to harmonics. As shown in Figure 2, the analytical model (using complex piezoelectric parameters, as discussed above, to take into account loss mechanisms) is able to accurately fit the experimental data over a wide frequency range. The accuracy is therefore comparable or better than the most onerous and complex conventional numerical models.

**Figure 3:**
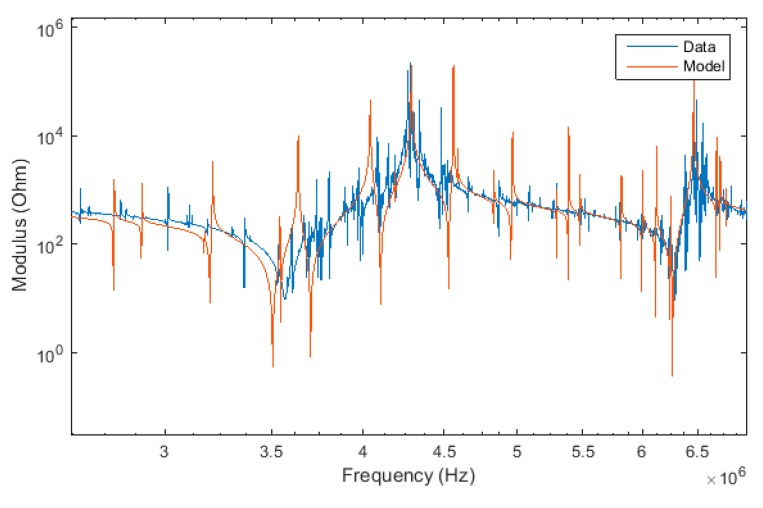
Impedance modulus spectrum of Y-cut LiNbO_3_ around the shear and the thickness resonance.

No resonances are found below 100 kHz, neither in the experimental data, nor in the model. All major resonances are correctly and simultaneously predicted by the model, some representative resonances of which are discussed below. For example, for the sample geometry chosen, Figure 3 shows the shear resonance at 3.5 MHz with its antiresonance at 4.3 MHz, as well as the thickness compression resonance around 6.3 MHz. Our assumption of the linear superposition of nine waves gives good results, although we do not predict all minor, higher order harmonics. Nevertheless, this is the first dynamic analytical model able to describe, at the same time, compression and shear modes in a piezoelectric material.

### 3.3. Experimental Observations Predicted by Analytical Model

A generalized dynamic analytical model to predict the electromechanical impedance spectrum of piezoelectric materials over a wide frequency range, from 20 Hz to at least 10 MHz was developed and an implementation of this model illustrated experimentally in the case of lithium niobate. Only very few assumptions are necessary: linear piezoelectric equations, linear superposition of mechanical vibrations, negligible leakage currents on the surface (but not necessarily inside the volume of the material), perfect metal electrodes, and an arbitrary but known geometry. In addition, no literature material parameters need to be inputted to use the model; quite the contrary, the dynamic analytical model can be used to extract almost any intrinsic material property from the impedance spectrum with only knowledge of the crystal symmetry of the material necessary.

**Figure 4:**
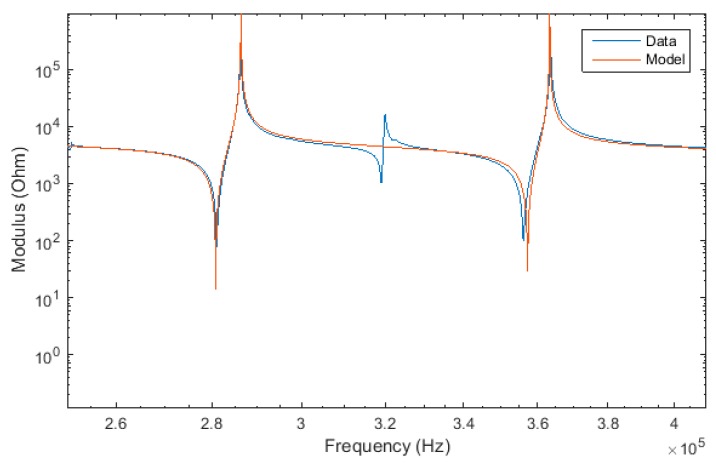
Impedance modulus spectrum of Y-cut LiNbO_3_ around the radial resonance.

Figure 4 shows two radial resonances, one with the two radial waves in-phase at 360 kHz and the other with the two radial waves out-of-phase at 282 kHz. The radial mode is composed of two waves with different velocities; this typically precludes traditional models from predicting the radial resonance at 282 kHz, although it is an important feature of the lithium niobate impedance spectrum. Although our model accurately predicts all important resonances, smaller resonances, such as the higher order secondary complex mode at 320 kHz, are not described. This is likely due to limitations of the linear superposition assumption. In addition, some minor harmonics are not predicted at the right frequency. This is probably due both to minor limitations in our model (particularly the linear superposition assumption), as well as imperfect experimental conditions (sample is not entirely mechanically free). Nevertheless, the analytical model shows an excellent prediction for the complex 3m crystal symmetry class, significantly better than can be obtained by conventional analytical models or phenomenological frameworks.

Shear-modes are analytically predicted and every predicted resonance can be experimentally observed, which is essential for accurately determining associated material coefficients. The model is computable even for a very complex piezoelectric material, such as lithium niobate of the 3m crystal symmetry class.

## 4. Conclusions

A generalized dynamic analytical model to predict the electromechanical impedance spectrum of piezoelectric materials was developed that can be used for any piezoelectric crystal symmetry class, no matter the orientation or sample geometry. We provide, as an illustration, how this model can be used to study a few representative resonance modes in lithium niobate, although naturally this model can be used to study any resonance mode desired. Thus, the model presented in this manuscript can be easily generated and exploited for the impedance spectroscopy resonance method. The impedance spectrum of a sample is a function of almost all intrinsic properties of the piezoelectric material, so that intrinsic material properties, including mechanical, electrical, and dielectric coefficients, can be readily and simultaneously extracted for any size crystal system, including at the nanoscale; the only prior knowledge required is the crystal class of the material. In addition, as this model is entirely based on first principles and can be used to study the effect of any applicable loss mechanism, it provides valuable insight into physical phenomena underlying the piezoelectric mechanisms inside these important and exciting materials.

## Figures and Tables

**Table 1:** Symbols used in the present article to describe the model.

a_i_	Half dimension of the sample along the direction x_i_ (m)	T	Time (s)
C_E_	Stiffness matrix under constant electric field (N/m^2^)	U	Displacement field (m)
C_D_	Stiffness matrix under constant dielectric displacement field (N/m^2^)	V	Electrical potential of the top electrode (V)
D	Dielectric displacement field (C/m^2^)	Z	Electrical impedance of the sample (Ω)
E	Electric field (V/m)	α	Wave number (rad/m)
e	Piezoelectric stress constant matrix (C/m^2^)	ε_S_	Permittivity under constant strain field (F/m)
h	Piezoelectric stress modulus matrix (N/C)	ε′	Permittivity’s real part (F/m)
j	Imaginary unit	ε″	Permittivity’s imaginary part (F/m)
Q	Electric Charge (C)	η_E_	Mechanical loss factor under constant electric field (N∙s/m^2^)
S	Strain tensor (1)	σ	Electrical conductivity (S/m)
T	Stress tensor (N/m^2^)	ω	Pulsation of the sample (rad/s)
